# Geneva Medical College

**Published:** 1855-09

**Authors:** 


*Geneva Medical College.*—We are requested to state that Prof. Wm. Sweetser has resigned the chair of Institutes and Practice of Medicine in this institution, and that Prof. Charles A. Lee is no longer connected with it.

